# Post-Thaw Non-Cultured and Post-Thaw Cultured Equine Cord Blood Mesenchymal Stromal Cells Equally Suppress Lymphocyte Proliferation *In Vitro*

**DOI:** 10.1371/journal.pone.0113615

**Published:** 2014-12-01

**Authors:** Lynn B. Williams, Laurence Tessier, Judith B. Koenig, Thomas G. Koch

**Affiliations:** 1 Department of Clinical Studies, University of Guelph, Guelph, Ontario, Canada; 2 Department of Biomedical Science, University of Guelph, Guelph, Ontario, Canada; 3 Department of Clinical Studies, Orthopaedic Research Lab, Aarhus University, Copenhagen, Denmark; Federal University of Rio de Janeiro, Brazil

## Abstract

Multipotent mesenchymal stromal cells (MSC) are receiving increased attention for their non-progenitor immunomodulatory potential. Cryopreservation is commonly used for long-term storage of MSC. Post-thaw MSC proliferation is associated with a lag-phase *in vitro*. How this lag-phase affect MSC immunomodulatory properties is unknown. We hypothesized that *in vitro* there is no difference in lymphocyte suppression potential between quick-thawed cryopreserved equine cord blood (CB) MSC immediately included in mixed lymphocyte reaction (MLR) and same MSC allowed post-thaw culture time prior to inclusion in MLR. Cryopreserved CB-MSC from five unrelated foals were compared using two-way MLR. For each of the five unrelated MSC cultures, paired MLR assays of MSC allowed five days of post-thaw culture and MSC included in MLR assay immediately post-thawing were evaluated. We report no difference in the suppression of lymphocyte proliferation by CB-MSC that had undergone post-thaw culture and MSC not cultured post-thaw (p<0.0001). Also, there was no inter-donor variability between the lymphocyte suppressive properties of MSC harvested from the five different donors (p = 0.13). These findings suggest that cryopreserved CB-MSC may have clinical utility immediately upon thawing. One implication hereof is the possibility of using cryopreserved CB-MSC at third party locations without the need for cell culture equipment or competencies.

## Introduction

Multipotent mesenchymal stromal cells (MSC) are receiving significant attention as a treatment option for various conditions. Currently MSC are thought to have either progenitor or non-progenitor cellular functions. Although studied extensively, progenitor cell integration into recipient tissues has only been observed in very small numbers challenging the importance of the MSC progenitor paradigm [1]–[3]. Di Nicola et al [4] observed MSC-mediated lymphocyte suppression occurred in a MSC-dose dependent, time independent, and reversible manner that did not require cell-to-cell contact *in vitro*. These findings resulted in a paradigm shift—from progenitor to non-progenitor functions—as a main mechanism by which undifferentiated MSC exerts therapeutic effect. Non-progenitor MSC actions that have been investigated include cell-to-cell fusion [5]–[7], organelle transfer [8],[9], reactive oxygen scavenge [10]–[13], and suppression of lymphocyte proliferation [4],[14],[15].

MSC-mediated lymphocyte suppression has been observed in equine *in vitro* studies. Expected equine lymphocyte proliferation following stimulation with either allogeneic lymphocytes or plant-based mitogens was suppressed by equine MSC derived from bone marrow, adipose tissue and umbilical cord blood (CB) *in vitro* [16]. Work in our lab confirmed the lymphocyte suppressive properties of equine CB-MSC [17]. One potential advantage of CB-MSC compared to other MSC sources is that they can be isolated and characterized prior to the donor sustaining and injury in case of autologous use. Allogeneic use of equine CB-MSC have also recently been reported in clinical cases without observed adverse reactions [18]. These reports suggest that allogeneic CB-MSC may have clinical utility as immune-modulatory agents.

Equine MSC lymphocyte suppression studies to-date have explored MSC that were maintained in culture prior to inclusion in so-called two-way mixed lymphocyte reactions (MLR) *in vitro*. Cryopreserved and passaged umbilical cord derived endothelial cells exhibit a proliferative lag-phase upon thawing and sub-culturing [19]. The lag phase however, was observed to be 36h longer for cryopreserved endothelial cells than non-cryopreserved cells. No difference was noted between fresh and cryopreserved/thawed human umbilical vein endothelial cells with regard to anti-inflammatory and anti-coagulant activity *in vitro*. The thawed cells, however, were allowed to overcome their proliferative lag phase following cryopreservation prior to inclusion in these *in vitro* assays [19]. Whether cryopreserved/thawed MSC exhibit an equivalent ‘functional lag-phase’ with regard to lymphocyte suppression is undetermined.

We hypothesized that *in vitro* there is no difference in lymphocyte suppression potential between post-thaw non-cultured (PTNC) equine CB-MSC immediately included in mixed lymphocyte reaction (MLR) and same MSC allowed post-thaw culture (PTC) prior to inclusion in MLR.

## Materials and Methods

### Ethics statement

This study was specifically approved by the University of Guelph Animal Care Committee with regard to the procedures of collection of equine peripheral blood lymphocytes and equine umbilical cord blood (animal use protocols 1756 and 1570). Additional research conducted using specimens of this kind does not require review by the Animal Care Committee (falls under CCAC Category of Invasiveness A) and therefore the mixed lymphocyte reactions can be considered to have been conducted in accordance with the institutional ethics guidelines. Collection of peripheral blood and cord blood was add-on procedures to the routine care of the horses. No animals were sacrificed during the study. Equine umbilical cord blood was collected on two privately owned commercial farms in Southern Ontario. Four of five samples were collected on one farm from Thoroughbred foals. One sample was collected on another farm from a Warmblood foal. Informed consent was obtained in writing from the horse owners/agents prior to sampling. The broodmares on the foaling farms are housed in large foaling boxes. Both farms are staffed 24/7 and mares are under constant video surveillance and carrying foaling alarms to allow for observed foaling and assisted delivery if needed. Umbilical cord blood was collected by the farm staff after receiving instruction by Dr. Koch. Instruction included video-review of cord blood collection. Cord blood was collected from an isolated segment of the umbilical cord after the umbilical cord had been clamped and detached from the foal. Peripheral venous blood was obtained from the Equine Research Herd owned by the Ontario Ministry of Agriculture and Food (OMAF). Once investigators have an approved animal care protocol from the University of Guelph Animal Care Committee access to these research horses are granted. In this study peripheral blood was collected from 5 adult mixed-bred horses. The adult horses on the research farm are housed in smaller groups with run-in sheds throughout the year. The horses are on pasture during the summer and during the winter they have access to large paddocks with gravel surface. Peripheral blood was collected by Drs. Williams or Koch. Collection of peripheral blood was collected under mild sedation (Xylazine HCl, 0.35–0.40 mg/kg bwt IV; Bayer, Toronto, ON) from the jugular vein following which manual pressure was applied for several minutes to aid hemostasis.

### Lymphocyte collection

Equine peripheral blood lymphocytes (PBL) were isolated from equine whole blood obtained from five unrelated adult horses. 450 mL of whole blood was collected into a commercially available blood collection bag containing anticoagulant (Na citrate) and processed within 2 hours of collection by Ficoll (GE Healthcare, Mississauga, ON) density gradient separation. 35 mL of whole blood was layered over 15 mL of Ficoll-Hypaque- plus (density 1.077 g/L) within a 50 mL conical tube. Samples were centrifuged at 500 x*g* for 30 minutes at room temperature (RT) with no brake. The interphase was collected and washed twice with phosphate buffered saline (PBS) following centrifugation at 500 x*g* for 10 minutes at RT. Washed PBL were suspended in 10 mL of MLR culture medium (MLR-CM) which consisted of Roswell Park Memorial Institute (RPMI 1640, Invitrogen, Burlington, ON) culture medium, 10% heat inactivated horse serum (Invitrogen, Burlington, ON), 1% penicillin/streptomycin (Invitrogen, Burlington, ON), and 1% L-glutamine (Lonza/Cambrex, Walkersville MD). Cells were counted using an automated fluorescent based cell counter (Nucleocounter NC-100, Mandel Scientific Company, Guelph, ON), re-suspended freshly prepared cryomedium (10% DMSO in MLR-CM) and frozen in 1.8 mL cryovials at a concentration of 6×10^6^ cells/mL. Cells were slowly frozen at a rate of −1°C/min to −80°C (Mr. Frosty, Nalgene, Mississauga, ON) before transfer for long-term storage in liquid nitrogen. At the time of use, the PBL were thawed in a 37°C water-bath, followed by centrifugation at 500 x*g* for 5 minutes at RT. The PBL were suspended in 5 ml MLR-CM, counted and adjusted to a concentration of 2×10^6^ PBL/mL.

### MSC collection, culture, and cryopreservation

Cryopreserved CB-MSC cultures from five unrelated foals (N = 5) were included. The CB-MSC cultures were established as previously described [20],[21]. These primary cell cultures were cryopreserved at passages ranging from P1–P3 and preserved at a concentration of 1×10^6^/mL. Same-batch MSC vials from each of the five foals were designated to one of two treatment groups—PTC and PTNC.

### MSC-PTC

Five days prior to MLR set-up, one CB-MSC cryovial from each foal was thawed in a 37°C water bath, slowly diluted into 5 mL of MSC culture medium (MSC-CM) consisting of Dulbecco's modified eagle medium containing 30% fetal bovine serum, 1% penicillin/streptomycin, and 1% L-glutamine. MSC suspensions were centrifuged at 300 xg for 5 minutes, supernatant removed and suspended in MSC-CM. An automated cell count was performed using an automated fluorescent based cell counter (Nucleocounter NC-100, Mandel Scientific Company, Guelph ON). Thawed MSC were seeded in polystyrene culture flasks at a density of 5,000 MSC/cm^2^ and incubated at 38°C, 5% CO_2_, in a humidified atmosphere for 5 days. MSC-CM was changed on day two and four of the incubation period. On the fifth day MSC were detached from the culture flasks using trypsin-EDTA and suspended in MSC-CM, and counted. Following centrifugation the MSC were suspended in MLR-CM at a concentration of 2×10^5^/mL.

### MSC-PTNC

On day five, the day of MLR set-up, the second same-batch cryovial from each foal was thawed. The MSC were suspended in MSC-CM as described above. MSC were counted and suspended in MLR-CM at a concentration of 2×10^5^/mL.

### MLR

Cryopreserved PBL were thawed in a 37°C water bath, slowly diluted into 5 mL of MLR culture media (MLR-CM). PBL suspensions were centrifuged at 300 x*g* for 5 minutes, the supernatant was removed, and cell pellet was suspended in MSC-CM. An automated cell count was performed using an automated fluorescent-based cell counter (Nucleocounter NC-100, Mandel Scientific Company, Guelph ON). PBL were subdivided into tubes for use as stimulator PBL and responder PBL, respectively. Stimulator PBL and CB-MSC were mitotically inactivated with 20Gy γ-radiation (Theratron 780C Cobalt 60, MDS Nordion, Ottawa ON). Irradiated stimulator PBL (PBLx) from all five horses were pooled in equal proportions at a concentration of 2×10^5^ PBLx/mL.

Non-irradiated responder PBL (PBL), PBLx, and irradiated MSC (MSCx) were combined in a 10∶1∶1 ratio, respectively, in 96 well round bottom plates and cultured five days at 38°C, 5% CO_2_, in a humidified atmosphere. Lymphocyte proliferation was determined by bromodeoxyuridine (Brd-U) assay, see below for details. A series of controls were prepared on each plate including: negative control (PBL + autologous PBLx), positive control (PBL + allogeneic pool of PBLx), Brd-U staining controls (PBL + FITC or 7-AAD in the absence of Brd-U), and unstained (PBL only). On the fifth day of MLR culture Brd-U was added to each well and cultured for an additional 24 hours before fixation and staining for flow cytometry.

MLR reactions were performed in triplicate reactions using responder PBL from three different horses; resulting in nine replicates of each reaction. Identical experiments were setup using MSC that had received 5 days of PTC or PTNC MSC. These paired MLR were cultured, processed, and evaluated simultaneously.

### Outcome assessment

On day 6 of MLR culture, cells were fixed and stained for Brd-U flow cytometry using a commercially available kit (BD Biosciences, Mississauga ON,) according to the manufacturers directions. In brief, MLR-CM was removed, cells were washed, fixed, and stained using FITC anti-BrdU antibody (to detect proliferative cells) and 7-AAD antibody (viability stain). After 24 hours, cells were analyzed by flow cytometry. The gate to identify resting and stimulated lymphocytes was maintained consistent throughout the experiments.

### Statistical Analysis

Raw data was imported into a statistical analysis software package (SAS, SAS institute, Cary, NC). A general linear model was used to analyze the effect of treatment (MSC cell line, positive/negative controls), lymphocyte donor, and post thaw culture period using the PROC MIXED function. All two and three way interactions were initially evaluated and non-significant effects and interactions were removed from the model. Residual analysis was performed in order to determine if ANOVA assumptions were met, to detect potential outliers, and evaluate the need for data transformation. The residuals were formally tested for normality using the four tests offered by SAS (Shapiro-Wilk, Kolmogorov-Smirnov, Cramer-von Mises, Anderson-Darling) and plotted against the predicted values and variables used in the model. For the purpose of determining statistical significance α was set at 0.05.

## Results

**Figure 1:**
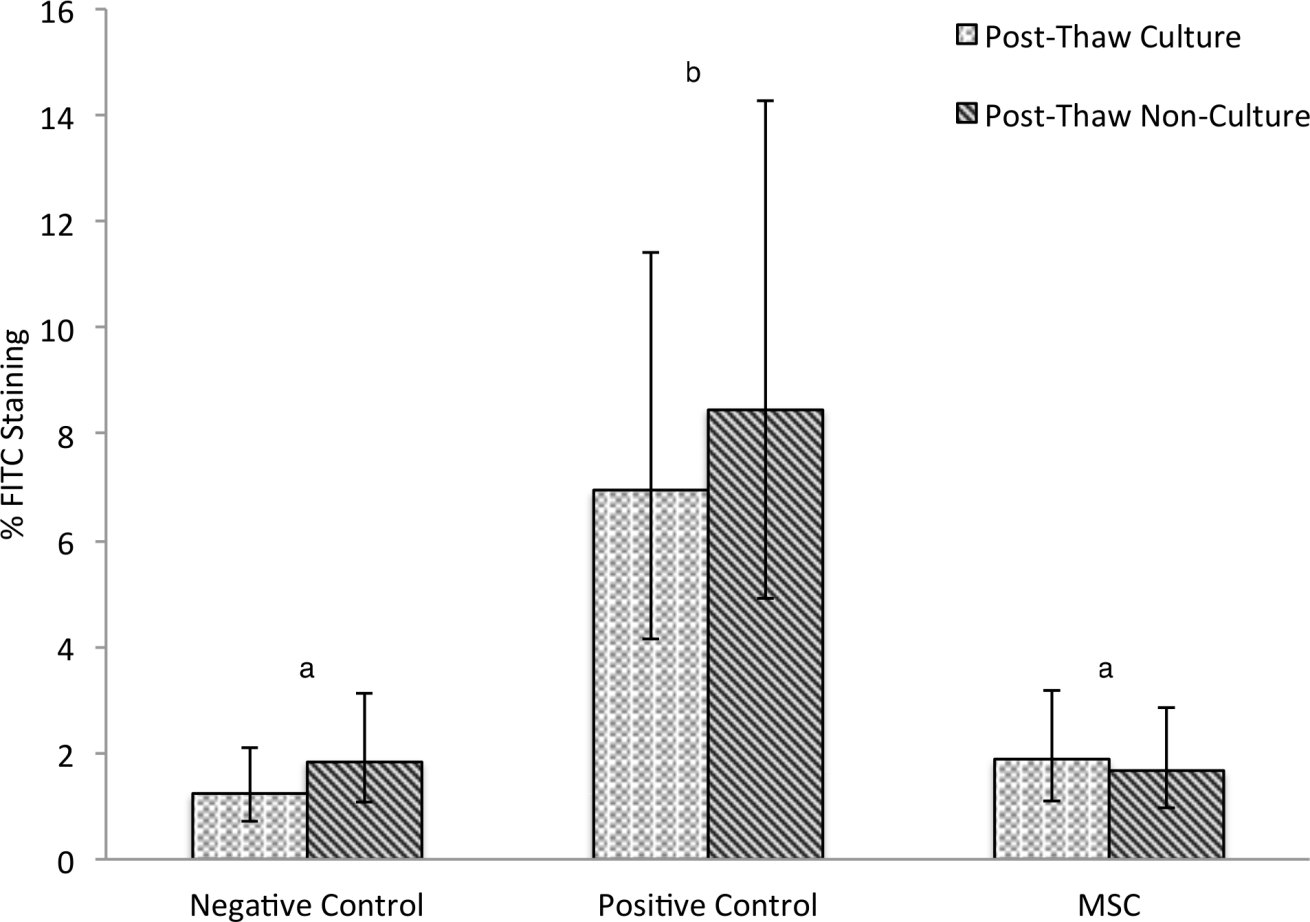
Mean FITC anti-BrdU staining following two-way MLR. Lymphocyte proliferation of un-stimulated (negative control) and allogeneic stimulated (positive control) compared to stimulated lymphocytes treated with MSC. Three biological replicates and three technical replicates were used in the negative control, positive control, and for evaluation of each of the five MSC cultures. Five thousand lymphocytes in each sample were observed and designated either FITC positive (proliferative in the previous 24 h) or FITC negative (not proliferative) Umbilical cord blood MSC from five cultures were either added after a 5-day post-thaw-culture period or identical MSC vials were thawed immediately prior to the experiment. No difference was observed between post-thaw-culture and post-thaw-non-culture groups. Different letters indicate statistically significant differences between means (α = 0.05). Error bars represent the 95% confidence interval.

**Figure 2:**
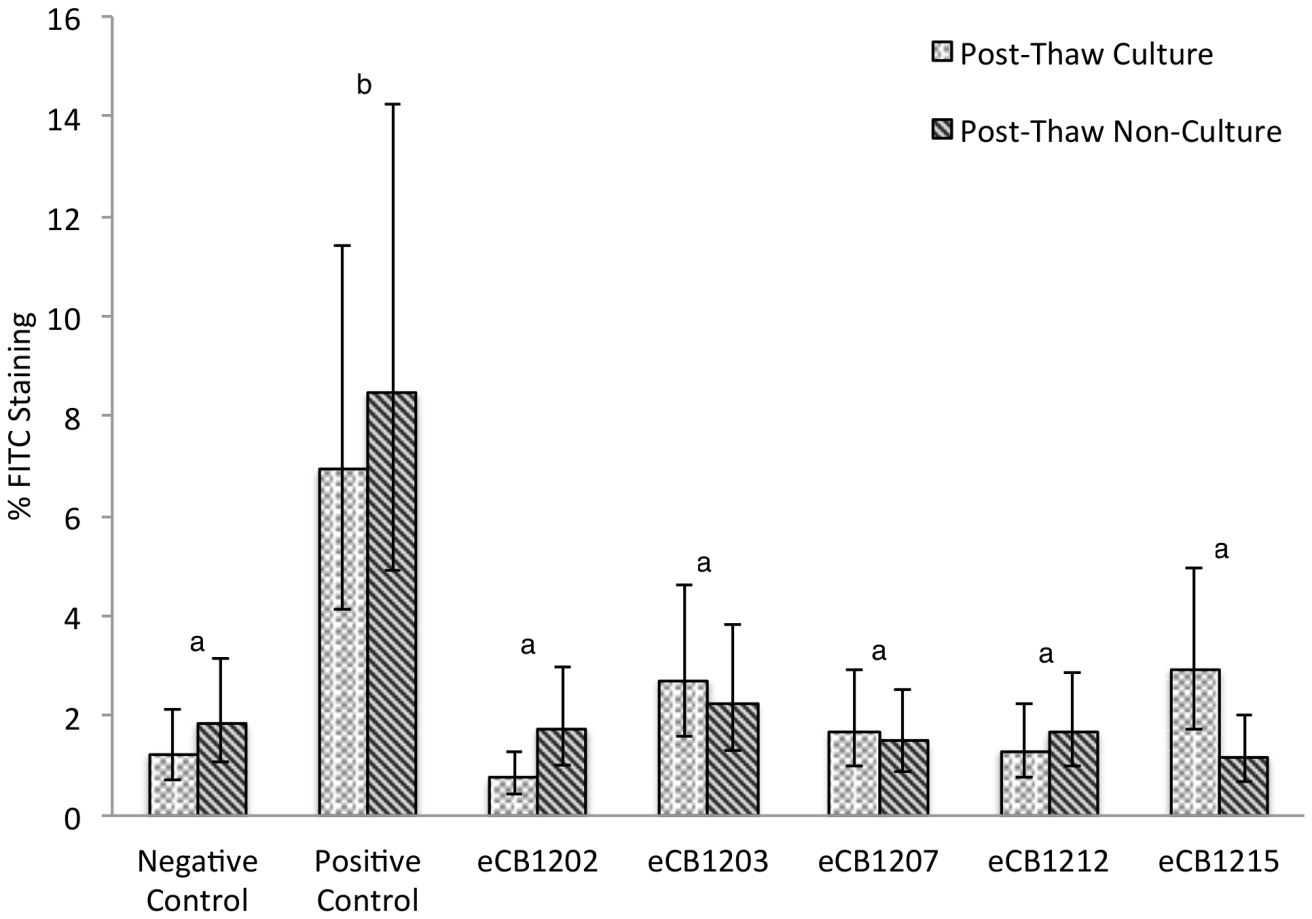
FITC anti-BrdU staining of five different CB-MSC cultures. Cryopreserved CB-MSC were either cultured five days or thawed immediately prior to MLR set-up. Each bar represents the mean percentage of FITC positive cells in nine replicates (triplicate wells for each of the three unrelated responder lymphocyte donors, 5000 lymphocytes evaluated in each sample). In no instance were significant differences observed between post-thaw-culture and post-thaw-non-culture groups. Different letters indicate statistically significant differences between means (α = 0.05). Error bars represent the 95% confidence interval.

**Figure 3:**
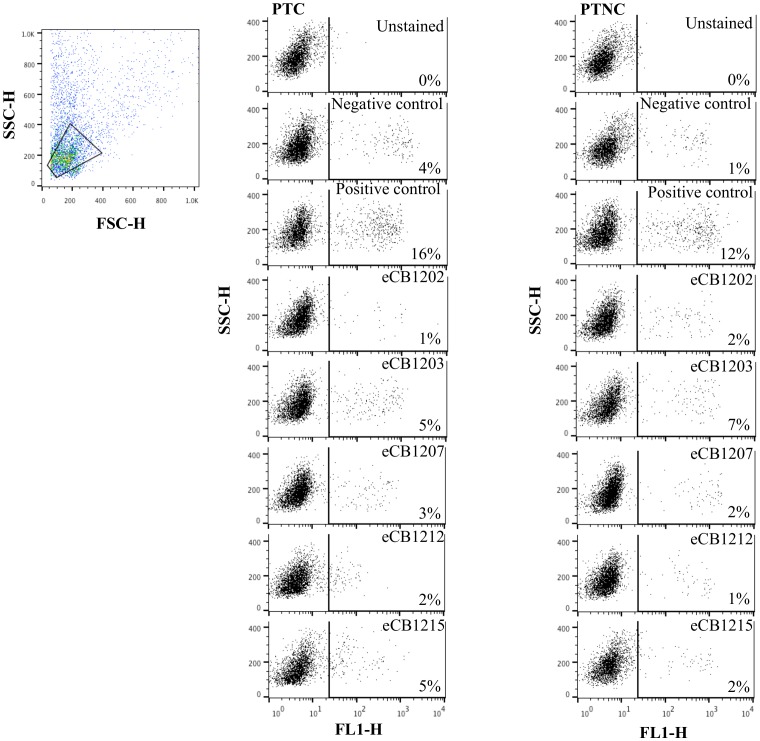
Cell proliferation during 2-way MLR was detected with a FITC-labelled antibody to BrdU. Dot plots represent fluorescence of lymphocytes identified by forward and side scatter after co-culture with post-thaw-cultured (PTC) and post-thaw-non-cultured (PTNC) CB-MSC. The percentage fluorescence (right quadrant) indicates the proportion of lymphocytes proliferating in autologous (negative control), allogeneic (positive control) and allogeneic plus CB-MSC reactions. Differences in lymphocyte proliferation induced by PTC and PTNC CB-MSC were not significant.

CB-MSC suppressed lymphocyte proliferation compared to the positive control, Figure 1 (p<0.0001). No difference was observed between CB-MSC cultures or between CB-MSC cultures and the negative control (p = 0.13). All MLR that included CB-MSC, no matter the post thaw status, proliferated significantly less than their associated positive control, Figures 2 and 3. Positive controls were always significantly different than negative controls (p<0.0001). The odds ratio of the positive control staining for Brd-U was 4.99 (95% CI 3.21-7.76) times the odds of any other treatment staining Brd-U positive.

## Discussion

Cryopreserved equine CB-MSC appear to constitutively suppress lymphocyte proliferation *in vitro* independent of post-thaw culture period. The noted homogeneity in the lymphocyte suppressive effects among CB-MSC suggests that although minor inter-donor variation did exist, screening of individual CB-MSC cultures prior to *in vivo* therapy may not be warranted.

Clinical use of cryopreserved CB-MSC immediately upon thawing would allow the attending veterinarian to determine treatment time independent of MSC procurement. Following injury, time is a critical factor as inflammatory mediators and cells quickly localize to the area of injury facilitating an inflammatory response [22],[23]. Despite the importance of inflammatory events in tissue healing, acute excessive inflammatory events or chronic inflammation is often associated with impeded tissue healing or further tissue damage [22],[24],[25]. This occurs as a result of activation of the host immune responses including elevated cytokine levels, reactive oxygen species, the release of host matrix metalloproteinases, and other collagenolytic enzymes [24].

Development of an “off the shelf” cryopreserved CB-MSC therapy appears feasible using an allogeneic strategy. Although lameness was not associated with IA MSC injection into healthy joints, mild inflammatory responses to IA injection of autologous and allogeneic MSC has been reported in the horse [3],[26]. Equine allogeneic MSC from CB and adipose tissue were not associated with adverse reactions when used as treatment for tendonitis in two small case series [18],[27]. The possible immune-privileged nature or immune evasive properties of MSC indicates potential for immediate MSC treatment using a cryopreserved allogeneic MSC product.

One caveat of the study is that PTNC CB-MSC were added into the reaction wells five days before evaluation of lymphocyte proliferation. We can therefore only conclude that any recovery period required by MSC prior to being lymphocyte suppressive is less than five days. In the initial report of lymphocyte suppression in MLR [4], the effect of human bone marrow-derived MSC was reported to occur independent of the time that MSC were added. Work with cryopreserved human MSC suggests recovery of normal cellular function occurs relatively quickly post-thaw [28],[29]. These studies investigated the effect of cold and osmotic shock associated with cryopreservation on human BM-MSC and concluded that the metabolic disturbances observed in thawed MSC ceased to exist after 24 h [28] and could be minimized using proper technique [29]. We speculate that the recovery period is short, and clinically irrelevant, as no difference in lymphocyte proliferation was observed. This notion is supported by the mean response we observed within CB-MSC cultures. In three of the five CB-MSC cultures evaluated, lymphocyte proliferation was less in the wells treated with CB-MSC that were from the PTNC group. In fact, of the two CB-MSC cultures where trends towards significant differences were observed within a sample pair, the sample with the lower mean had been treated with CB-MSC from the PTNC group.

In conclusion, our findings suggest that cryopreserved equine CB-MSC may have clinical utility immediately upon thawing. One implication hereof is the possibility of using cryopreserved CB-MSC at third party locations without the need for cell culture equipment or competencies.
